# Pronunciation difficulty, temporal regularity, and the speech-to-song illusion

**DOI:** 10.3389/fpsyg.2015.00048

**Published:** 2015-01-29

**Authors:** Elizabeth H. Margulis, Rhimmon Simchy-Gross, Justin L. Black

**Affiliations:** Music Cognition Lab, University of ArkansasFayetteville, AR, USA

**Keywords:** speech-to-song illusion, repetition, music and language, music perception, meter

## Abstract

The speech-to-song illusion (Deutsch et al., 2011) tracks the perceptual transformation from speech to song across repetitions of a brief spoken utterance. Because it involves no change in the stimulus itself, but a dramatic change in its perceived affiliation to speech or to music, it presents a unique opportunity to comparatively investigate the processing of language and music. In this study, native English-speaking participants were presented with brief spoken utterances that were subsequently repeated ten times. The utterances were drawn either from languages that are relatively difficult for a native English speaker to pronounce, or languages that are relatively easy for a native English speaker to pronounce. Moreover, the repetition could occur at regular or irregular temporal intervals. Participants rated the utterances before and after the repetitions on a 5-point Likert-like scale ranging from “sounds exactly like speech” to “sounds exactly like singing.” The difference in ratings before and after was taken as a measure of the strength of the speech-to-song illusion in each case. The speech-to-song illusion occurred regardless of whether the repetitions were spaced at regular temporal intervals or not; however, it occurred more readily if the utterance was spoken in a language difficult for a native English speaker to pronounce. Speech circuitry seemed more liable to capture native and easy-to-pronounce languages, and more reluctant to relinquish them to perceived song across repetitions.

## INTRODUCTION

Music and speech offer excellent comparative cases to illuminate the mechanisms subserving human communication (cf. Patel, 2008). They share many acoustic features, but differ in salient ways too – music tends to feature slower pitch changes, more stable fundamental frequencies, and rhythmic structure that gives rise to the perception of an isochronous beat. Music and speech may share not only developmental origins (McMullen and Saffran, 2004), but also common evolutionary origins (Wallin et al., 2001), yet they often seem quite phenomenologically distinct. It can seem that music is heard as music, and speech is heard is speech, and that is that. Several years ago, however, Deutsch et al. (2008) reported a striking illusion where repeating a segment of speech could engender a perceived transformation from speech to song. In this illusion, participants first hear an ordinary spoken utterance. Then they hear a segment from this utterance repeated 10 times in succession. Finally, they rehear the original utterance, but on this hearing, the segment that had been repeated sounds as if it were being sung rather than spoken. Rhythmic and pitch content comes strikingly to the fore, and this change in perceptual orientation results in a change in the category to which listeners attribute the stimulus.

Since the discovery of this illusion, various studies have sought to examine what qualities must be in place for this perceptual transformation to occur. Deutsch et al. (2011) showed that no illusory change to song occurred if the repetitions were inexact – if they were slightly differently transposed in pitch on each repetition, or if the syllables were jumbled into different orderings on each repetition. Tierney et al. (2013) were able to collect a set of spoken utterances that tended to transform to song after repetition, and a set of spoken utterances that did not tend to transform. The utterances that did transform were distinguished from the others by slightly more stable fundamental frequency contours within syllables, and by more regular spacing of inter-accent intervals. When speech was perceived as song, regions associated with pitch processing such as the superior temporal gyrus and regions associated with auditory-motor integration such as the precentral gyrus were differentially activated. These results suggest that not only does a shift from speech to song reflect increased attention to pitch, but it might also entail more imagined motor involvement. When we hear a song, we tend to sing along in our heads in a way that is quite different from how we listen to speech.

Falk et al. (2014) showed that when the utterance’s pitch contour was made up of stable tonal targets, people perceived the transformation to song earlier and more frequently. Rhythmic aspects of the utterance did not play as big of a role. They also manipulated the regularity of the pause between utterances, but found it had no effect on the speech to song transformation. These findings are consistent with Mantell and Pfordresher (2013), which used a vocal imitation task to show that people could replicate the absolute pitch of song more accurately than the absolute pitch of speech, but there was no difference in accuracy between song and speech on replication of timing. People with and without formal musical training experienced the illusion the same way.

Given the increased auditory-motor integration for song perception revealed in Tierney et al. (2013), we wondered whether part of what distinguishes attending to music from attending to speech is a participatory stance, where the listener begins to sing through a tune in her head while it is playing after she has heard it a few times – a hypothesis explored in Margulis (2013). To address this hypothesis, languages of varying pronunciation difficulty were used. It should be easy to imaginatively reproduce native language speech after a few repetitions, but progressively harder as the language gets more difficult to pronounce relative to the native language. For example, since Catalan might be judged by English speakers to be easier to pronounce than Hindi, a few repetitions of a Catalan sentence might allow English speakers more accurate auditory imagery of the phrase than a few repetitions of a Hindi sentence, resulting in a stronger tendency for the Catalan sentence to transform to music. This hypothesis suggests, then, that the differences between pre and post repetition speech-to-song ratings should be greatest in the native language (English), and progressively smaller as the languages get more difficult to pronounce.

We also wondered whether the higher-level temporal regularity produced by spacing the repetitions at identical intervals was necessary for the illusion to occur. Falk et al. (2014) found that temporal regularity was not necessary, but we used a different method for making the repetitions temporally irregular, a method that made the difference between the regular and irregular versions more salient. We sought to confirm that higher-level temporal regularity was not required for repetition to transform speech into song.

Our study used recordings from an archive of native speakers telling the same story in different languages as stimuli. Half of the recordings were from languages hypothesized to be easier for English speakers to pronounce, and half were from languages hypothesized to be harder for English speakers to pronounce. The English language recording of the story was also included for comparison. Half of the participants heard these recordings in a temporally regular condition, where each repetition followed after an identical temporal interval, and half of the participants heard them in a temporally irregular condition, where the repetition occurred at unpredictable intervals. They rated a phrase from each utterance on a 5-point scale from speech to song both before and after the repetitions. The difference in ratings was taken as an index of the transformation from speech to song. At the end of the session, participants responded to various questions about the languages in the study, including how difficult each might be to pronounce, so that the results could be interpreted in terms of participants’ actual ratings of pronunciation difficulty, in addition to the hypothesized categories.

## MATERIALS AND METHODS

### PARTICIPANTS

The 24 participants (8 male, 16 female) ranged in age from 18 to 22 with a mean age of 19.6 years (*SD* = 1.2). In exchange for participating, they received extra credit in a general music appreciation course aimed at non-majors called Music Lecture. Only one participant reported being enrolled as a music major. Only six of the participants reported formal training in music; all of it at a young age and all of it short lived. Thus, unlike Deutsch et al. (2011), which used musically trained listeners as participants, this study focuses predominantly on people without formal musical training. Since results did not change when the one music major participant was excluded, we retained all participants in the reported analyses. All participants were native English speakers, and all reported normal hearing.

One participant reported fluency in each of the following languages: Vietnamese, Japanese, Chinese, and Swedish. 12 participants reported some experience with Spanish. Of these, three reported they were fluent, three reported their level of Spanish ability to be advanced, three reported it to be at the beginner level, and the rest reported an intermediate ability. One participant had studied beginning Japanese, and one participant reported proficiency in Vietnamese. The Chinese speaker, the Japanese speaker and one Spanish speaker reported using the language in childhood. None of the participants reported receiving training in any of the languages used in the experiment.

All participants signed an informed consent form before starting the experiment. The protocol was approved by the University of Arkansas Institutional Review Board.

### MATERIALS

Seven excerpts from non-tonal languages were selected from the examples used in the Handbook of the International Phonetic Association (1999), available at http://web.uvic.ca/ling/resources/ipa/handbook_downloads.htm. Each excerpt consisted of a person speaking the following utterance “The north wind and the sun were disputing which was the stronger, when a traveler came along wrapped in a warm cloak. They agreed that the one who first succeeded in making the traveler take his cloak off, should be considered stronger than the other” in one of seven languages: English, Catalan, Portuguese, French, Croatian, Hindi, or Irish. Aside from English, three languages (Catalan, Portuguese, and French) were hypothesized to be easier for English speakers to pronounce, and three languages (Croatian, Hindi, and Irish) were hypothesized to be harder for English speakers to pronounce. All the languages except for Catalan were spoken by a female.

The mean utterance length was 12.1 s (*SD* = 2.7). For each language, a segment was extracted from the utterance using Audacity 2.0.3. The segment extraction was made at about the three-quarter mark of each utterance. The mean segment length was 2.7 s (*SD* = 0.3).

Two stimuli were created for each language: a temporally regular and a temporally irregular version. The regular versions consisted of the full utterance followed by 10 segment repetitions, each separated by 1000 ms. The irregular versions were more complex, consisting of the full utterance followed by 10 segments, each separated by time intervals that were random percentages (between 1% and 50%) shorter and longer than 1000 ms. For each randomly selected percentage, one interonset interval was created by shortening the 1000 ms span the appropriate amount, and another was created by lengthening it. To increase the salience of the temporal shifts, 400 ms was subtracted from each of the sub-1000 ms values, and added to each of the over-1000 ms values. For example, the randomly selected percentage 15% generated the interonset intervals 450 ms (850–400 ms) and 1550 ms (1150 ms + 400 ms).

A total of 10 interonset intervals were generated from 5 randomly selected percentages. The order of the 10 time interval lengths was randomized. In a few cases, this randomization resulted in two similar time intervals placed back to back (e.g., 510 ms followed by 520 ms); when this happened, one of the time intervals was moved to a different position in the sequence. The object was to create a series of time intervals that made the extraction of meter as unlikely as possible. One advantage of the procedure is that the total duration of all the repetitions was the same in the regular and the irregular condition, eliminating an explanation based on exposure length rather than temporal regularity.

### PROCEDURE

Participants were seated at a computer terminal in a WhisperRoom 4′ by 4′ Enhanced, Double Wall Isolation Booth and outfitted with Sennheiser HD 600 headphones. Instructions were presented on screen and stimuli were presented over the headphones. Participants made all responses using the keyboard and mouse.

Participants were randomly assigned to one of two groups. Group one heard the repetitions in temporally regular form; group two heard the repetitions in temporally irregular form. All other procedures for the two groups were the same.

First, participants answered a series of demographic questions. Next, they performed the task for each of the 7 languages, with the language order randomized. For each language, they were told to listen carefully to an utterance. After the utterance was complete, they were told they would hear a segment from the utterance and be asked to rate it on a scale from 1 to 5, with 1 signifying “sounds exactly like speech” and five signifying “sounds exactly like singing.” They were played the segment, and asked to rate it. Next, they were told they would rehear the utterance, followed by 10 repetitions of the segment, followed by a restatement of the entire utterance. It was explained that they should then rate how the segment sounded within that utterance on the same 1 to 5 scale. Thus, participants rated the segment twice—once before and once after the repetitions. Finally, participants answered a series of questions about each of the languages in the experiment, with the language order randomized. For each language, they were replayed the utterance and asked to enter the name of the language. Next, they rated the familiarity of the language on a scale from 1 to 5. Finally, they were asked how easy they thought it would be to pronounce the words in the language accurately, on a scale from 1 to 5.

## RESULTS

**FIGURE 1:**
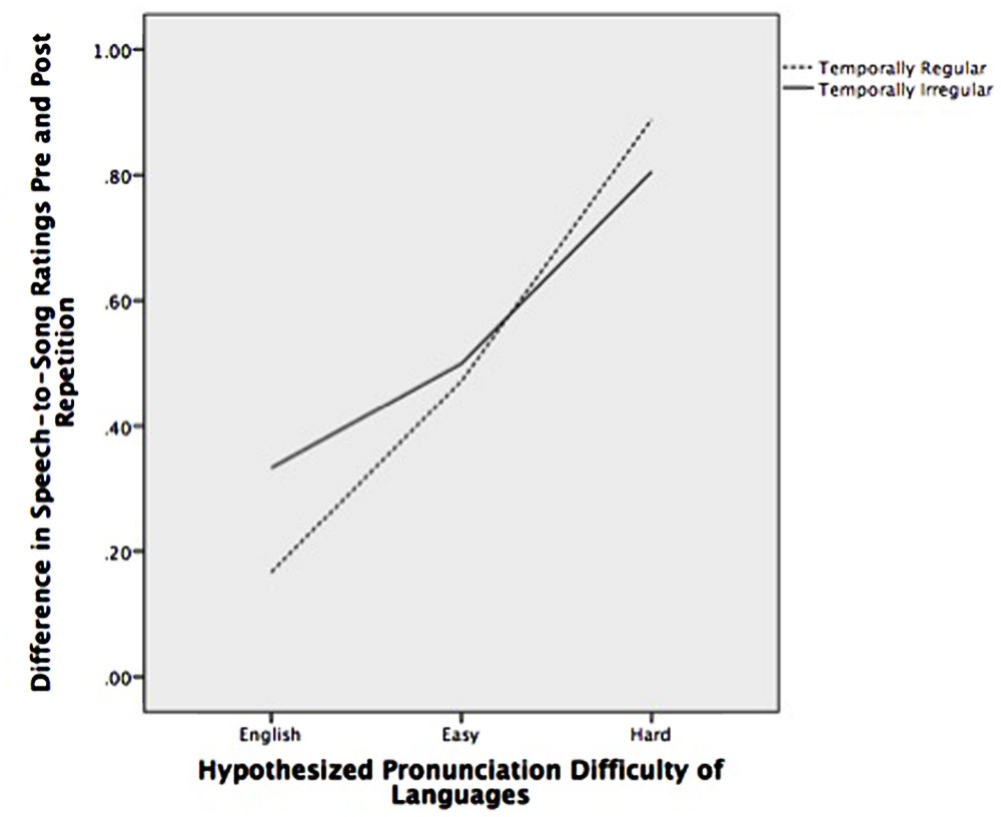
**Difference in speech-to-song ratings before and after repetition for temporally regular and irregular stimuli by hypothesized pronunciation difficulty of languages**.

A linear mixed model was used with the difference in speech–song ratings pre and post repetition as the dependent variable, language difficulty (native, easy, and hard) and temporal structure (regular vs. irregular) as fixed factors and language (English, Catalan, Portuguese, French, Croatian, Hindi, Irish) as a repeated variable. As shown in **Figure 1**, there was a main effect of the hypothesized pronunciation difficulty of the language on the change in speech–song ratings, *F*(2,140) = 6.45, *p* = 0.002; however, there was no main effect of temporal regularity *F*(1,27) = 0.03, *p* = 0.87.

**Table 1** shows the speech–song ratings for each language category before and after the repetitions. The pre and post repetition ratings were different for every category except English, signifying that a transformation from speech to song occurred in every foreign language, but not the native one. Rating changes from pre to post repetition increased from the native to easy to hard categories, signifying an intensification of the speech-to-song illusion for languages hypothesized to be difficult for native English speakers to pronounce.

**Table 1:** Mean speech-to-song ratings for each language difficulty category before and after repetition.

Language difficulty	Repetition	Mean	SE	Rate change (post–pre)	Pairwise comparison, pre to post, using sidak adjustment for multiple tests
Native	Pre	1.08	0.18	0.25	*p* = 0.17
	Post	1.33	0.18		
Easy	Pre	1.38	0.11	0.48	*p* < 0.001
	Post	1.86	0.11		
Hard	Pre	1.56	0.11	0.84	*p* < 0.001
	Post	2.40	0.11		

**Table 2** shows the mean rating change for each language in the temporally regular and irregular condition. Patterns were broadly similar between the two groups, with easier to pronounce languages engendering less dramatic transformation from speech to song and harder to pronounce languages engendering more dramatic transformation. **Table 3** summarizes this effect for each of the three language difficulty categories.

**Table 2:** Mean changes in speech-to-song rating from pre to post repetition for each language in each condition.

Temporal structure	Language	Mean rating change
Regular	English	0.17
	Catalan	0.42
	Portuguese	0.25
	French	0.75
	Croatian	0.92
	Hindi	0.83
	Irish	0.92
Irregular	English	0.33
	Catalan	0.33
	Portuguese	0.50
	French	0.67
	Croatian	0.67
	Hindi	0.75
	Irish	1.00

**Table 3:** Mean changes in speech-to-song rating from pre to post repetition for each category in each condition.

Temporal structure	Language difficulty	Mean rating change
Regular	English	0.17
	Easy	0.47
	Hard	0.89
Irregular	English	0.33
	Easy	0.50
	Hard	0.81

**FIGURE 2:**
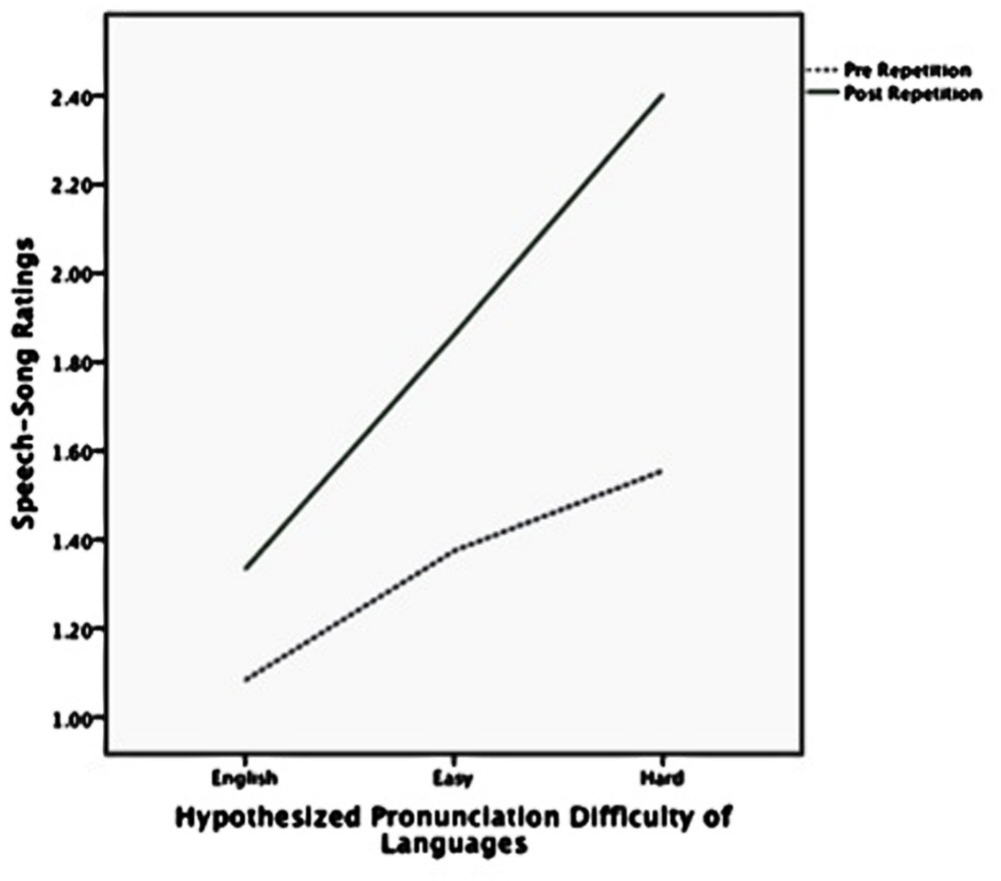
**Speech-song ratings pre and post repetition by hypothesized pronunciation difficulty of languages**.

As shown in **Table 4**, participants rated how difficult they thought each language would be to pronounce accurately. Participants’ ratings generally correlated with the hypothesized difficulty ratings, but their judgments tended to group into four categories rather than three – Native (English); Easy (Catalan, Portuguese); Medium (French, Croatian); and Hard (Hindi, Irish). In the hypothesized categories, French was grouped with Easy and Croatian with Hard. The data were reanalyzed using these four categories rather than the original three as predictors. Rating change varied significantly according to the difficulty of each language as rated by the participants; *F*(3,138) = 5.30, *p* = 0.002, as shown in **Figure 2**. **Table 5** lists the means for these categories.

**Table 4:** Participants’ difficulty and familiarity ratings for each language compared with their hypothesized categories.

Language	Participants’ difficulty rating	Participants’ familiarity rating	Hypothesized difficulty categories	Participants’ difficulty categories
English	1.38	4.58	Native	Native
Catalan	2.75	3.38	Easy	Easy
Portuguese	3.08	2.79	Easy	Easy
French	3.50	3.17	Easy	Medium
Croatian	3.50	2.83	Hard	Medium
Hindi	4.13	2.29	Hard	Hard
Irish	4.04	2.46	Hard	Hard

**Table 5:** Mean speech-to-song ratings for each of the participants’ language difficulty categories.

Participants’ difficulty categories	Repetition	Mean	Rate change (post–pre)	Pairwise comparison, pre to post, using sidak adjustment for multiple tests
Native	Pre	1.08	0.25	*p* = 0.17
	Post	1.33		
Easy	Pre	1.27	0.37	*p* = 0.04
	Post	1.64		
Medium	Pre	1.48	0.75	*p* < 0.001
	Post	2.23		
Hard	Pre	1.65	0.87	*p* < 0.001
	Post	2.52		

**FIGURE 3:**
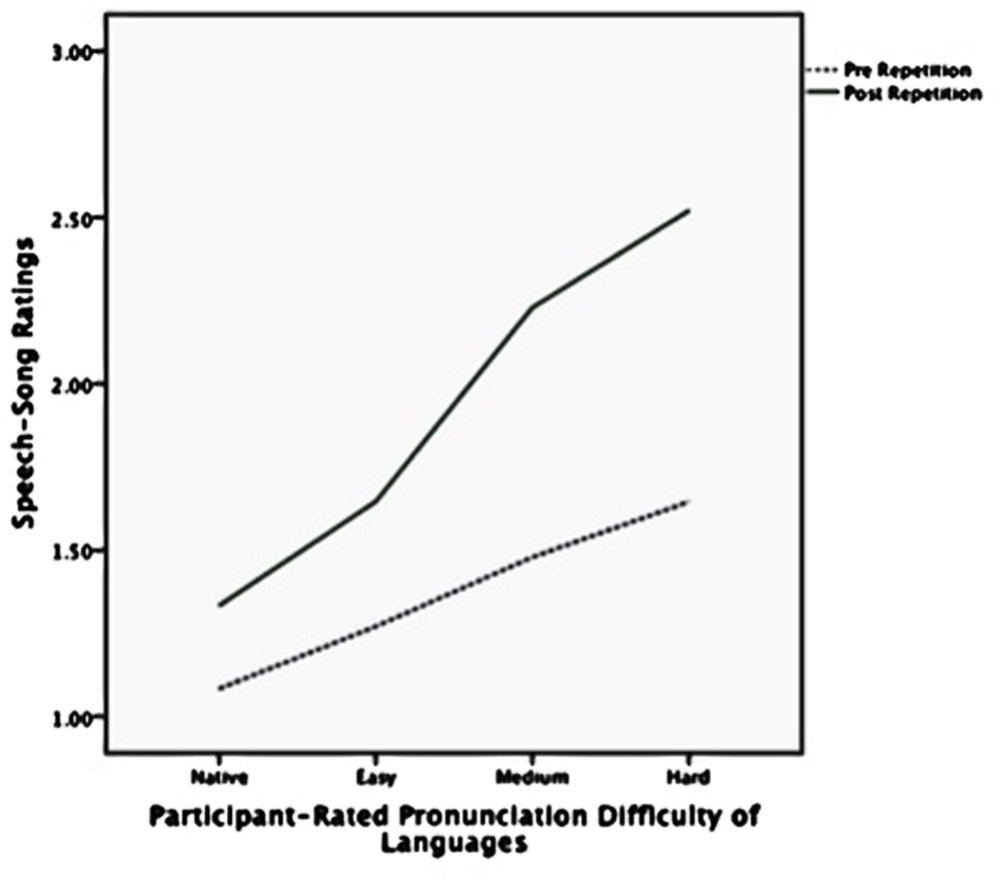
**Speech-song ratings pre and post repetition by participant-rated pronunciation difficulty of languages**.

**Figure 2** shows the speech–song ratings before and after the repetition for each of the three hypothesized language difficulty categories. **Figure 3** shows the same trend for speech–song ratings within each of the four participant-rated language difficulty categories. For each breakdown of the categories, harder-to-pronounce languages were rated as more songlike to begin with; however, harder-to-pronounce languages also experienced a larger speech-to-song transformation than easier-to-pronounce languages. The native language experienced the least transformation, the easy and medium more, and the hard the most.

Participants also rated the familiarity of each language (also shown in **Table 4**). These ratings were marginally predictive of speech–song rating changes post repetition, *F*(1,155) = 3.80, *p* = 0.05.

Ninety-six percent (all but one) of the participants correctly identified the English language. Eighty-three percent correctly identified the French language. Every other language was correctly identified by one participant (4% of respondents), except Portuguese, which was correctly identified by 4 (16%). A large percentage of participants misidentified Catalan as Spanish, potentially accounting for the high familiarity ratings for Catalan despite the low success with identifying its name. This pattern underscores the distinction between perceived pronunciation difficulty and mere familiarity; the sound of the French language was quite familiar to participants, and most were able to identify it correctly; however, they still rated the language as moderately difficult to pronounce. Responses did not differ by gender.

In order to ascertain whether there was something inherently more music-like about stimuli in some categories, we used two measures from Tierney et al. (2013), one to assess the degree of fundamental frequency stability across syllables in each utterance, and the other to assess the degree of temporal regularity among syllable stresses. Following the procedure outlined in that paper, we first used Praat to assess the fundamental frequency stability across each syllable in each utterance, by calculating the average fundamental frequency change in semitones per second. In Tierney et al. (2013), utterances that were more likely to transform to song had more within-syllable fundamental frequency stability (less change). **Table 6** lists the mean fundamental frequency change for each syllable in each of the seven languages. These means do not vary significantly between pronunciation difficulty categories, except between the Native and Easy participant-rated categories. Stimuli in the category rated by participants as Easy exhibited more fundamental frequency variability per syllable than stimuli in the Native category. If acoustic characteristics were driving the effect, we would expect to see the stimuli with less intrasyllable frequency variability (the Native stimuli) transform to song more easily; however, the opposite effect occurred. This reinforces the notion that the pronunciation difficulty, rather than some more basic acoustic characteristic, influenced the degree to which particular utterances were susceptible to the speech-to-song illusion.

**Table 6:** Intrasyllable fundamental frequency change for each language.

Language	Mean intrasyllable fundamental frequency change	SD
English	16.61	13.38
Catalan	31.70	13.24
Portuguese	36.34	19.24
French	19.04	10.53
Croatian	32.61	17.24
Hindi	20.52	5.78
Irish	18.09	6.43

Next, following another procedure in Tierney et al. (2013), we identified the timepoints of the onsets of stressed syllables in each utterance. To assess the temporal regularity of the speech segment, we measured the SD of the duration between successive onsets of stressed syllables. The results for each language are shown in **Table 7**.

**Table 7:** Variability of duration between stressed syllable onsets for each language.

Language	SD of durations between stressed syllable onsets
English	0.07
Catalan	0.13
Portuguese	0.18
French	0.07
Croatian	0.16
Hindi	0.11
Irish	0.13

If the results were driven by these acoustic characterizations rather than by pronunciation difficulty, we would have expected to see fundamental frequency change correlate negatively with the size of the speech–song rating change across repetitions; languages with large intrasyllable fundamental frequency changes (lower frequency stability) should transform to song less easily, as shown by smaller speech–song rating changes. Instead, no consistent pattern emerged (*p* > 0.05). We would also have expected to see the standard deviations of stressed syllable onsets vary negatively with speech–song rating change across repetitions; more temporally irregular utterances should transform to song less easily. Again, however, no consistent pattern emerged (*p* > 0.05).

## DISCUSSION

Contrary to our initial hypothesis, utterances spoken in languages more difficult to pronounce relative to the listener’s native tongue were actually *more* susceptible to the speech-to-song illusion. Since it should have been easier to imaginatively simulate the pronunciation of the syllables in easier to pronounce languages, it seems on first pass that this kind of virtual participation must not be essential to musical attending. Yet there is another way of understanding this result.

The pre-repetition ratings from this experiment show that harder to pronounce languages *started out* sounding more musical to listeners, even before any repetitions had contributed the illusory transformation. When the data are reanalyzed using the same methods except substituting initial ratings rather than rating differences as the dependent variable, there is a main effect of hypothesized language difficulty, *F*(2,40) = 5.05, *p* = 0.008. If the speech-to-song illusion had been independent of the pronunciation difficulty, the solid lines on **Figures 2** and **3** would have moved up in parallel to the dotted lines, signifying that languages in each of the categories transformed to song after repetition to roughly the same degree. But instead the slope of the solid lines is steeper; the languages that were more difficult to pronounce, and more songlike to start with, became *even more* songlike after repetition than did the easier to pronounce languages. This suggests that when speech circuitry captures acoustic input, it is more resistant to releasing it to other perceptual mechanisms. Speech circuitry seems more likely to capture acoustic input when it is easy to pronounce than when it is hard to pronounce.

To imagine what this release might entail, consider the semantic satiation effect (Severance and Washburn, 1907). It is normally very difficult to perceive a word independently of its semantic correlate. It takes many repetitions before the meaning starts to disintegrate and the sounds can be heard on their own terms. Across the course of these repetitions, it is almost possible to feel the release as the lexicon’s grip on the word recedes. The harder to pronounce languages may not have elicited as strong a grip by language regions in the first place, allowing repetition to effect a starker shift to song.

Our results supported those in Falk et al. (2014) showing that the illusion occurred whether the repetitions were spaced regularly or irregularly. Temporal regularity does not seem to be a necessary factor in the speech-to-song illusion. The illusion seems to be driven by repetition itself rather than by the emergence of larger-scale temporal regularity.

Additionally, the transformation to song does not seem to be driven by the acoustic characteristic of fundamental frequency stability within syllables, or the acoustic characteristic of regularity between stressed syllable onsets. This strengthens the case that pronunciation difficulty—and perhaps associatedly, the degree to which an utterance is captured by speech circuitry—can influence any particular utterance’s susceptibility to the speech-to-song illusion.

Because the speech-to-song illusion exposes a border between the perception of language and the perception of music, it is especially useful for illuminating how different aspects of acoustic input get emphasized in different contexts. Listeners may start with more acute perception of the prosody and songlike aspects of foreign languages, especially if they are very difficult to pronounce relevant to their native tongue. The more closely acoustic input conforms to the sounds of their native language, the tighter a grip the language circuitry may have on that input, and the less accessible language-irrelevant (or less language relevant) aspects of the sound may be.

To return to the initial hypothesis, although the harder-to-pronounce languages may have been difficult to imaginatively *speak* along with, they might have actually been easier to imaginatively *sing* along with. If language circuitry was less dominant in the processing of utterances in these languages, it may have been easier to disregard formant transitions and tune into the prosodic contour and timing of the pitch changes, features that are already more traditionally musical. Future work might examine people’s capacity for vocal imitation in languages relatively easier or harder to pronounce, similar to Mantell and Pfordresher (2013), to investigate this hypothesis.

## Conflict of Interest Statement

The authors declare that the research was conducted in the absence of any commercial or financial relationships that could be construed as a potential conflict of interest.
